# Well‐Switching to Reduce Arsenic Exposure in Bangladesh: Making the Most of Inaccurate Field Kit Measurements

**DOI:** 10.1029/2021GH000464

**Published:** 2021-12-01

**Authors:** Yusuf Jameel, M. Rajib Hassan Mozumder, Alexander van Geen, Charles F. Harvey

**Affiliations:** ^1^ Department of Civil and Environmental Engineering Massachusetts Institute of Technology Cambridge MA USA; ^2^ Lamont‐Doherty Earth Observatory Columbia University Palisades NY USA; ^3^ Now at Gradient Boston MA USA

**Keywords:** arsenic, well‐switching, field kit, Bangladesh, public health

## Abstract

Well‐switching programs in Bangladesh have successfully lowered arsenic exposure. In these programs, households switch from wells that are labeled “unsafe” to nearby wells labeled “safe,” but these designations are usually based on inherently inaccurate field kit measurements. Here, we (a) compare the efficacy of field‐kit measurements to accurate laboratory measurements for well switching, (b) investigate the potential impact on well switching of the chosen “safe” threshold, and (c) consider the possible benefits of providing more detailed concentration information than just “safe” and “unsafe.” We explore different hypothetical mitigation scenarios by combining two extensive data sets from Araihazar Bangladesh: a blanket survey of 6595 wells over 25 km^2^ based on laboratory measurements and 943 paired kit and laboratory measurements from the same area. The results indicate that the decline in average arsenic exposure from relying on kit rather than laboratory data is modest in relation to the logistical and financial challenge of delivering exclusively laboratory data. The analysis further indicates that the 50 μg/L threshold used in Bangladesh to distinguish safe and unsafe wells, rather than the WHO guideline of 10 μg/L, is close to optimal in terms of average exposure reduction. We also show that providing kit data at the maximum possible resolution rather than merely classifying wells as unsafe or safe would be even better. These findings are relevant as the government of Bangladesh is about to launch a new blanket testing campaign of millions of wells using field kits.

## Key Points

- Well‐switching based on kit or laboratory measurements produces similar reductions in arsenic exposure
- The optimal well switching threshold for reducing arsenic exposure is near 50 μg/L, higher than the WHO guideline of 10 μg/L
- Providing actual kit readings instead of classifying wells as just "unsafe" or "safe" could lead to a higher reduction in arsenic exposure

## 1 Introduction

### 1.1 Background

In rural Bangladesh, and South Asia more generally, treated drinking water distributed through a piped system is rare. Sadly, millions drink arsenic‐contaminated groundwater from their household well. An estimated 45 million people in Bangladesh are exposed to arsenic concentrations greater than 10 μg/L, the World Health Organization (WHO) guideline, causing more than 100,000 excess spontaneous abortions and infant and adult deaths every year (Flanagan et al., 2012; Quansah et al., 2015).

Well testing conducted over a decade ago has induced millions of households to switch to nearby safe wells (Jamil et al., 2019; van Geen et al., 2002). Implicitly, the success of this program has been recognized by the government as it is about to launch a new wave of well testing across much of the country. The well‐switching approach is viable because low‐arsenic wells are often in proximity to contaminated wells and many households make the extra effort of fetching water from another well. Well‐switching programs require measurements of arsenic concentration in most wells across a community but, unfortunately, most wells in Bangladesh are untested, partly due to the continuing installation of new wells (van Geen et al., 2014). The number of groundwater wells in Bangladesh has increased steadily (Dey et al., 2017; Jamil et al., 2019) and on the order of >1 million wells/year continue to be installed (van Geen et al., 2014). In most villages, only a small minority of wells are tested (George et al., 2012; Jamil et al., 2019).

To fill this data gap, the government of Bangladesh has announced a several hundred‐million‐dollar project to test groundwater arsenic concentration across the country using inexpensive field kits. Field kits are used in Bangladesh instead of more accurate laboratory methods that require more resources: transport of the samples to laboratories, expensive spectrometers, and return of the results to well owners. Field kits can be performed on‐site by local people with basic training. However, field kits are less accurate than spectrometric measurements conducted in laboratories and provide only categorical measurements representing nominal ranges (e.g., 50–100 μg/L). A recent comparative analysis between several types of kits concluded that improved precision and accuracy are necessary to employ kits for health‐related decision making (Reddy et al., 2020). In contrast, it has been argued that existing kits have been effective in identifying arsenic contaminated wells (Ahmed et al., 2006; van Geen et al., 2002, 2005). Testing all wells in Bangladesh, including new wells that continue to be installed, in the laboratory is unrealistic.

This article focusses on two key questions concerning well‐switching based on field kits. First, how and to what extent do the inaccuracies of field kits diminish the effectiveness of well switching. Second, what arsenic concentration threshold, if any, should be used to switch from a high arsenic well to a well that is lower in arsenic. In Bangladesh, a single threshold of 50 μg/L arsenic—which is higher than the WHO guideline of 10 μg/L—is used to categorize wells as either safe or unsafe. In 1993, when the WHO guideline for safe level of arsenic in drinking water was reduced to 10 μg/L, Bangladesh did not reduce its drinking water standard (Smith & Smith, 2004). To the best of our knowledge, no previous study has considered what concentration threshold produces the best outcome for a program of well switching. This question is important because the Bangladeshi government is spending millions of dollars on field test kits to implement well switching, and because millions of people may switch their water supply based on these measurements.

Here, we compare the effectiveness of well switching recommendations based on accurate spectrometric‐based (also referred as laboratory) and less accurate kit‐based measurements using data from Araihazar Bangladesh. Our analysis capitalizes on the strength of the Araihazar data set which includes accurate laboratory measurements of arsenic from 6595 wells with precisely know locations and 943 dual measurements made by both accurate laboratory methods and field kits in a subsequent study in the same area. Because this data set is so extensive, we have accurate knowledge of the arsenic in nearly all wells over a large area and so we avoid making assumptions about the distribution or spatial pattern of arsenic concentrations. We first calculated the conditional probabilities of the nominal kit categories for wells with arsenic concentration between 0 and 1,000 μg/L using the 943 paired kit‐laboratory measurements. Then, we simulated nominal kit categories for accurately measured arsenic in 6595 wells in the same region 20 years ago (van Geen et al., 2003). The spatial distribution of arsenic, lateral and vertical, in groundwater of this region is remarkably similar to that in the country overall (BGS/DPHE, 2001).

### 1.2 The Subtleties of Well‐Switching

**Figure 1:**
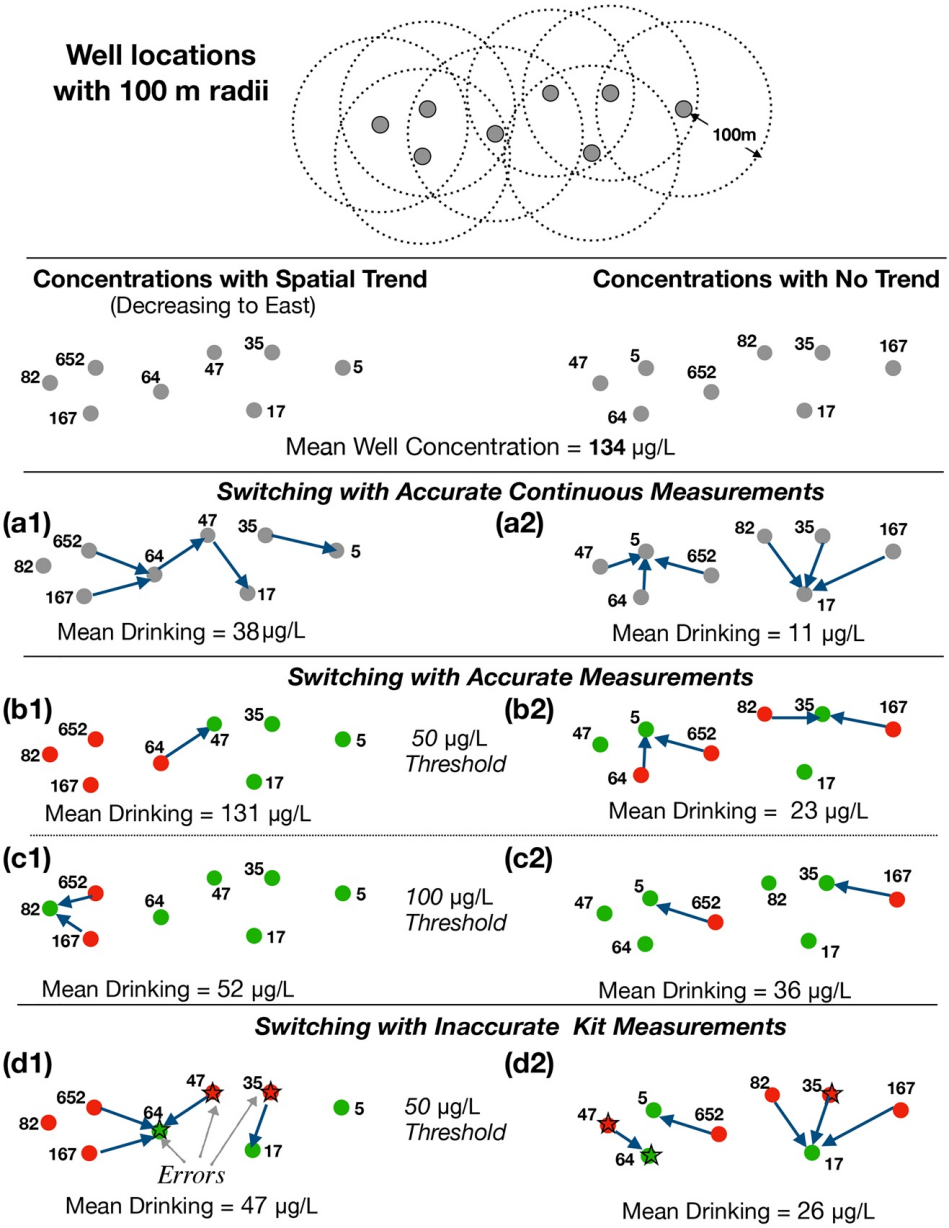
Schematic of a hypothetical group of wells and their arsenic concentrations that illustrates well switching patterns. Top panel: Positions and arsenic concentrations for eight hypothetical wells (shown in gray circle) and the 100‐m radii in which switching is possible. First column: Examples where arsenic concentrations are autocorrelated in space, generally decreasing to the east. Second column: Examples where arsenic concentrations are uncorrelated in space. Row A: The ideal base case where switches are based on accurate continuous arsenic measurements. Rows B and C: Switches are based on thresholds. Row D: Switches are based on kit measurements that mis‐assign some wells to the wrong category. In row D, incorrectly categoriezed wells due to field‐kit errors are shown in star.

The efficacy of well switching for lowering exposure depends in surprising ways on the spatial distribution of well arsenic, the threshold set for safe and unsafe wells, and the accuracy of testing methods. We illustrate these complexities with a hypothetical but representative set of eight wells (Figure 1). This hypothetical example demonstrates the problems that motivate our analysis of the Araihazar data, a far larger data set. We consider two patterns of arsenic concentrations: one with concentrations that are spatially correlated so that there is a decreasing trend to the east; the other, with the same set of concentration values, but rearranged to be uncorrelated across the domain. Both examples use the same set of eight arsenic concentrations, just arranged differently, and hence create the same exposure before well switching, but produce very different exposures after people switch to nearby wells with lower arsenic concentrations (Figures 1a–1d). This example demonstrates how different well switching programs can affect arsenic exposure and how different measurement methods and different safe/unsafe concentration thresholds affect arsenic exposure after switching.

First, we consider well switching for the ideal situation in which all arsenic concentrations are perfectly known and every participant switch to the well with the lowest arsenic concentration within 100 m (Figures 1a1 and 1a2). This ideal, but unrealistic, switching scenario serves as an upper bound to which we compare more realistic scenarios with uncertain measurements and discrete thresholds. In the ideal case, where arsenic concentrations are spatially correlated and generally lower to the east (Figure 1a1), well switching drops the mean concentration of consumed water from 134 to 38 μg/L. In this case, three of the eight households “chain switch”; they switch to wells whose owners themselves switch to wells with even lower concentration, a behavior that may be unrealistic. When there is no spatial correlation (Figure 1a2), there is no such chain switching and groups of households instead switch in clusters to a well with locally low concentrations, reducing the mean consumed concentration to 11 μg/L, an even lower value than the case where concentrations are spatially correlated.

Categorizing wells precludes switching within a category. Consequently, switching opportunities are lost when wells are categorized into “safe” above a threshold and “unsafe” below that threshold (Figures 1b and 1c). For example, when the threshold is 50 μg/L (Figure 1b1), the wells with 652 and 167 μg/L do not switch to the well with 64 μg/L because all of three of these wells are in the “unsafe” category above 50 μg/L. For this hypothetical arrangement of wells, a well switching program with a threshold value of 50 μg/L is therefore much less effective than the ideal case that uses continuous concentrations (Figure 1a1). The mean arsenic concentration is reduced only to 131 μg/L from 134 (Figure 1b1), rather than 38 μg/L as in the case when continuous concentrations were used. There is also no chain switching when there are only two categories.

In the next example, we demonstrate that a different threshold value can lead to different outcomes of well switching (Figures 1c1 and 1c2). In Figure 1c1, a threshold of 100 μg/L produces a much lower average consumed concentration than for a 50 μg/L threshold as in b1 because the well with 82 μg/L is now labeled safe and the two wells with the highest concentrations (652 and 167 μg/L) switch to this well. In contrast, a comparison of Figures 1b2 and 1c2 show how the outcome produced by 100 μg/L threshold can be worse than for a 50 μg/L threshold because the wells at 84 and 62 μg/L are now categorized as safe, and don't switch to wells with lower arsenic concentrations. The example proves that the optimal threshold value is not necessarily the concentration that has been deemed safe to drink based on health or other criteria. It raises an important question concerning well switching: what is the optimal threshold for categorizing wells as “safe” or “unsafe” to minimize arsenic exposure?

In the last part of this hypothetical example, we demonstrate how field‐kit errors that lead to incorrect categorization also led to different well‐switching outcomes. In Figure 1d1, the mislabeling of a 64 μg/L well as safe, even though it is over the 50 μg/L threshold, surreptitiously leads to a better outcome. Because this well is mislabeled (due to an inaccurate field‐kit measurement), neighboring households with even higher concentration wells now switch to it, and the overall mean consumed concentration falls from 134 to 47 μg/L, in fact much lower than the 131 μg/L for accurate measurements (Figure 1b1). In Figure 1d2, the mislabeling of 47 μg/L well as unsafe leads to switching to a mislabeled well with higher concentration (64 μg/L) as safe. The mislabeling as well as the lack of spatial gradient leads to a higher reduction in kit‐based arsenic switching from 134 to 26 μg/L in example 1d2. Perhaps counterintuitively, in this case, less accurate kit results lead to a greater reduction in arsenic exposure compared to accurate lab measurements.

### 1.3 Scope of the Analysis

In this article, we analyze a large set of field data to statistically characterize outcomes for different well switching strategies and answer the questions demonstrated by the hypothetical examples above. We use arsenic concentrations measured in the laboratory across several thousand wells in Araihazar as our test data set. We supplement this with another set of field kit data from the same area paired with laboratory measurements. The Araihazar area has been the focus of many previous studies including behavioral studies that analyzed how household decisions to switch wells depend on information about well arsenic concentrations (Bennear et al., 2013; Huhmann et al., 2019; Madajewicz et al., 2007) and has been the home to the extensive Health Effects of Arsenic Longitudinal Study (HEALS) (Ahsan et al., 2006). The site was also the locus of many geochemical studies of arsenic in groundwater, more specifically the vulnerability of low arsenic aquifers to contamination (Mihajlov et al., 2016; Mozumder et al., 2020). We focus here on questions that are important for designing a well‐switching program: What are the probabilities of assigning correct (and incorrect) color placards to a well where arsenic concentrations are measured by field kits? In other words, how often do less accurate field‐kit data lead to either a failure to correctly label a contaminated well or mis‐categorization of a safe well as unsafe? How and to what extent does the inaccuracy of kit data diminish the effectiveness of well switching? If a threshold has to be used, what is the optimal value distinguishing “safe” and “unsafe” wells that minimizes exposure? How does the spatial pattern of arsenic concentrations impact the effectiveness of well switching?

## 2 Methods

### 2.1 Data Sets

**Figure 2:**
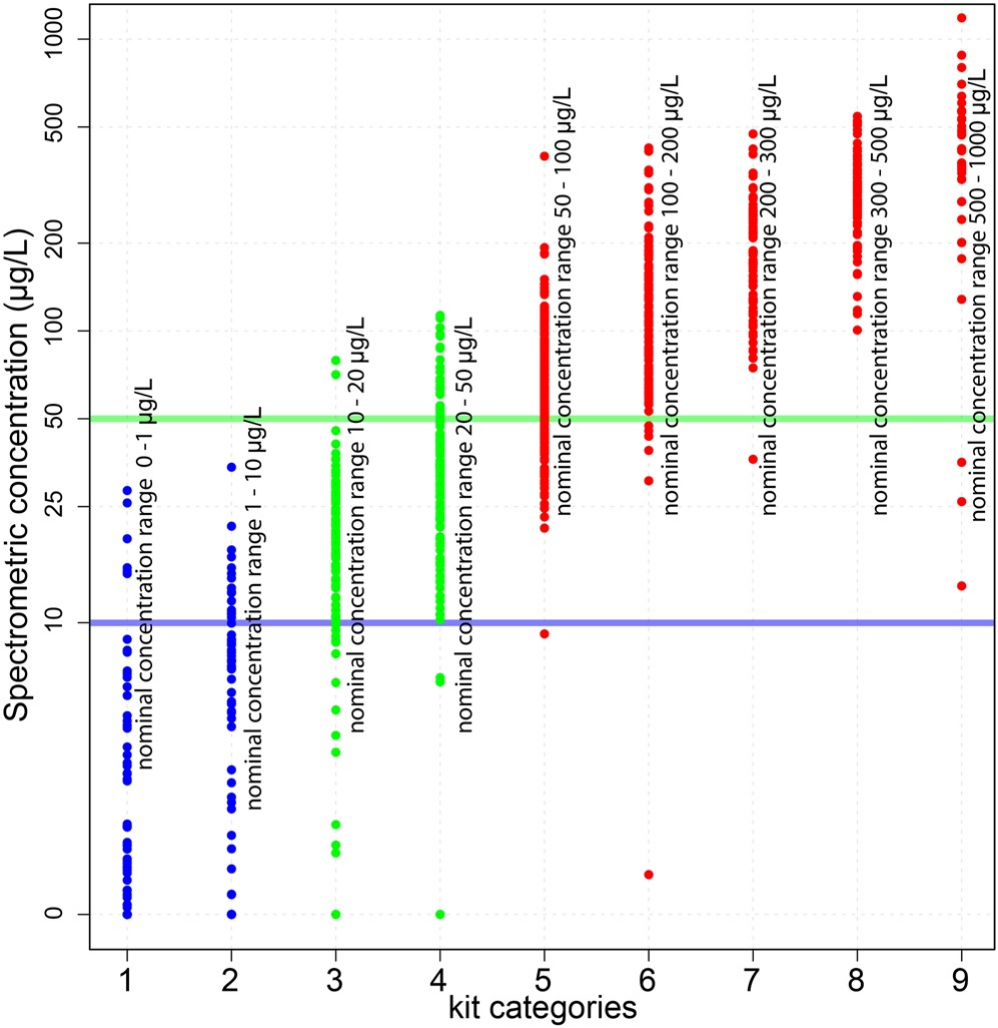
Distribution of spectrometric arsenic concentrations (*y*‐axis) within kit categories (*x*‐axis) for the 943 wells with paired spectrometric and kit measurements. Kit categories shown in blue, and green are classified as uncontaminated and kit categories shown in red are classified as contaminated. The 10 and 50 μg/L threshold are shown in blue and green lines. The data clearly show that the true arsenic range for any nominal category exceeds the range allotted to it.

**Figure 3:**
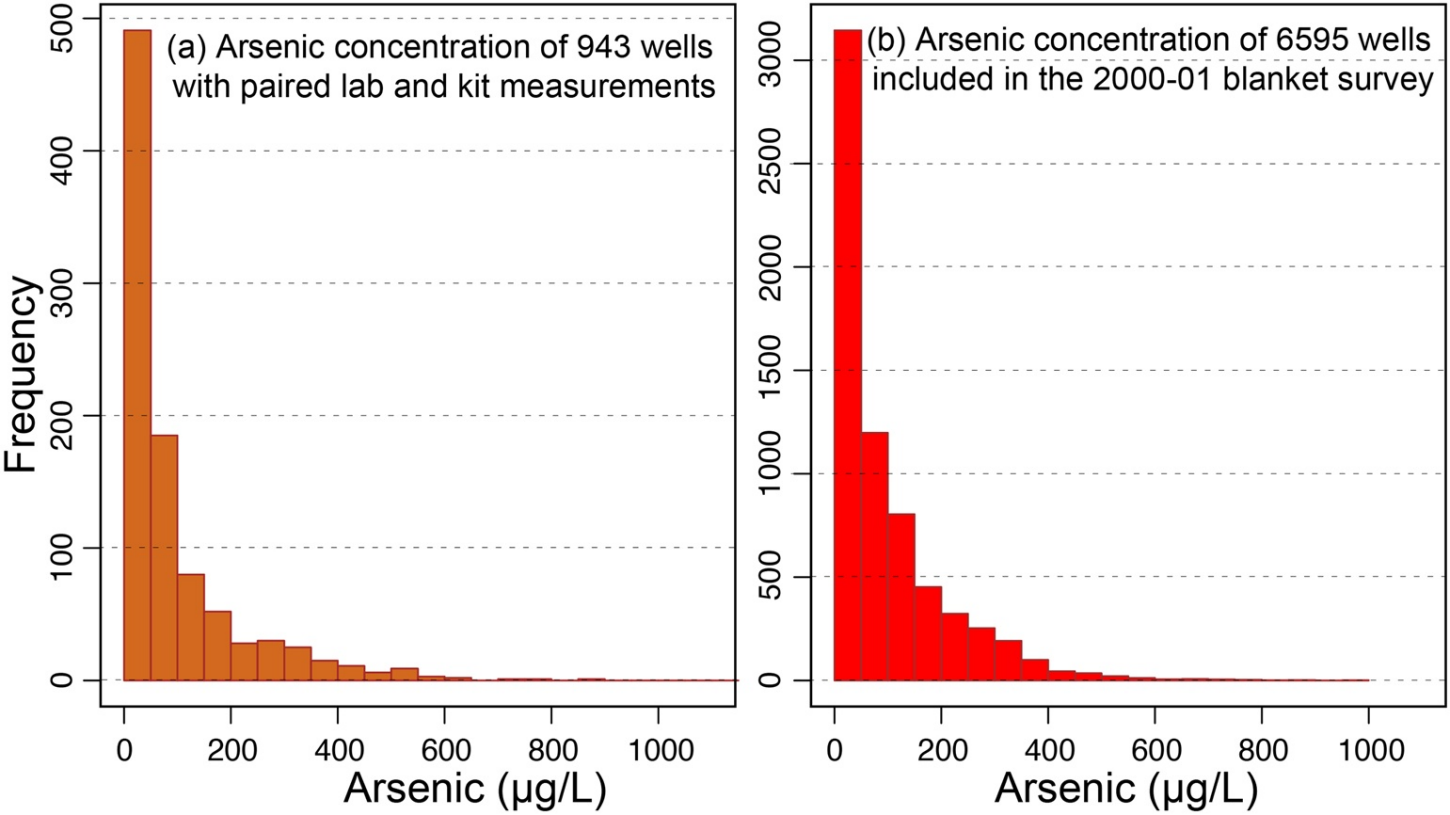
Distribution of laboratory measured arsenic concentration: (a) 943 wells with paired laboratory and kit measurements and (b) 6595 wells surveyed in 2000–2001.

Two data sets previously collected as a part of the HEALS program in Araihazar Bangladesh provide the necessary data for our analysis. The first set pairs field kit measurements of arsenic concentration conducted in 2012–2013 with accurate measurements made by inductively coupled plasma mass spectrometry (ICPMS) for 943 different wells (van Geen et al., 2014). This pairing enables a statistical assessment of errors in kit measurements. The ICPMS measurements have ±5% relative errors (Cheng et al., 2004). Field kit measurements are categorical, where each of nine different categories nominally represents a range of arsenic concentration (e.g., 10–50 μg/L). We refer to these ranges as nominal because the actual concentrations often fall outside the range (Figure 2). The second data set contains accurate measurements from 6595 wells representing every well within a 25‐km^2^ region that were sampled in 2000–2001 (van Geen et al., 2003). This large data set provides both the density of data to represent neighboring well concentrations and the extent of data to analyze a large‐scale well switching program. The distribution of groundwater arsenic concentration in both the data sets are statistically similar (two‐sample Kolmogorov‐Smirnov [K‐S] test, *p* = 0.02, Figure 3). We can avoid making assumptions or using parametric models to describe the arsenic distribution and spatial pattern because of the size and extent of these data sets.

### 2.2 Statistical Characterization of Field Kit Errors

We characterize the errors made in placing wells in safe and unsafe categories by analyzing the data set of paired kit and spectrometric measurements from 943 wells in Araihazar (Figure 2). Government programs apply green or red paint to the pump head to indicate if wells are safe or unsafe. However, in a kit‐based testing of all of Araihazar upazila conducted in 2012, three colors were used for longer‐lasting placards: blue for arsenic <10 μg/L, green for arsenic between 10 and 50 μg/L, and red for wells with arsenic >50 μg/L.

**Figure 4:**
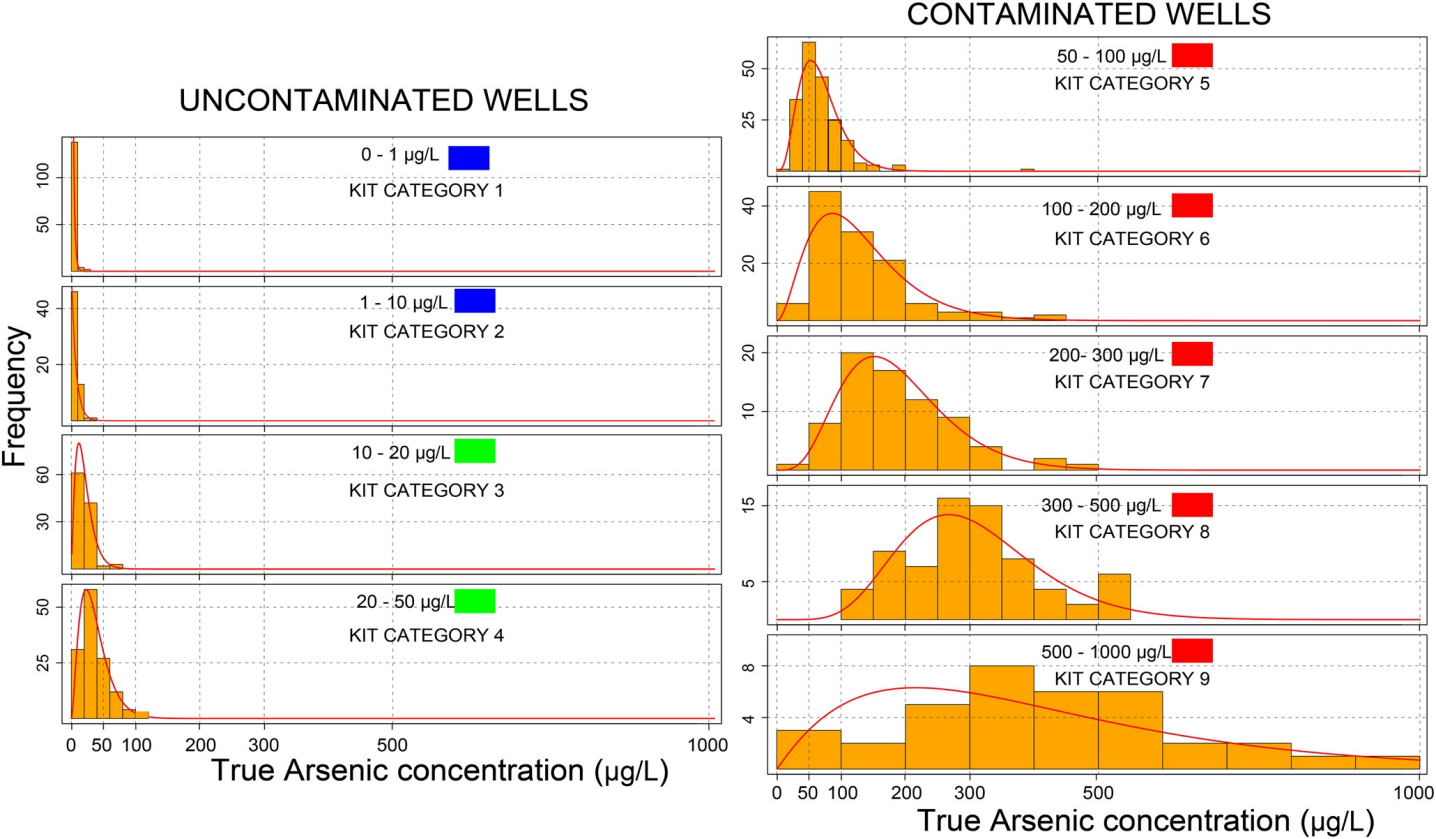
Frequency distribution (orange bars) of the arsenic concentration (*x*‐axis) for the respective nominal kit categories for the paired data set with 943 measurements. The red line is the best fit parametric distribution to the data for each kit categories. The placards posted on well based on the kit categories are also shown in each panel. Wells with blue and green placards are considered as uncontaminated and wells with red placards are considered as contaminated.

To calculate the probabilities of an incorrect label, we first estimate a probability density function $\;f_{n}\left(\theta \right)$ for the actual concentrations (θ) within each kit category *n*. We take the laboratory‐measured concentrations associated with each kit category and fit a parametric probability distribution function (Figure 4). We evaluated multiple distributions including gamma, normal, Weibull, and exponential and chose the best fit using the K‐S test. We then used these nine (for each of the kit category) probability density functions to calculate the conditional probabilities of assigning a particular category conditioned on the spectrometric arsenic measurements:

(1) $$P\left(\text{Kit}\;\text{category}\;=n|\;\text{Arsenic}\;=\;\theta \right)\;=\frac{f_{n}\left(\theta \right)}{\sum_{1}^{9}f_{i}\left(\theta \right)}$$

where P(Kitcategory=n|Arsenic=θ) in Equation 1 provides the probability of observing each kit category if the laboratory measured concentration is θ and *i* corresponds to the nine nominal kit categories. Table 1 provides a worked‐out example for calculating the conditional probabilities of the different kit categories for a well with arsenic concentration of 100 μg/L.

**Table 1:** Density and Conditional Probabilities of the Nominal Kit Categories for the Laboratory (Spectrometric Measured) Concentration of 100 μg/L

Nominal kit categories	Density	Conditional probabilities
Kit category 1 (nominal range 0–1 μg/L)	<0.0001	*p*(kit category = 1 | As = 100 μg/L) < 0.001
Kit category 2 (nominal range 1–10 μg/L)	<0.0001	*p*(kit category = 2 | As = 100 μg/L) < 0.001
Kit category 3 (nominal range 10–20 μg/L)	<0.0001	*p*(kit category = 3 | As = 100 μg/L) < 0.001
Kit category 4 (nominal range 20–50 μg/L)	<0.0001	*p*(kit category = 4 | As = 100 μg/L) < 0.001
Kit category 5 (nominal range 50–100 μg/L)	0.005	***p*(kit category = 5 | As = 100 μg/L) = 0.3**
Kit category 6 (nominal range 100–200 μg/L)	0.008	***p*(kit category = 6 | As = 100 μg/L) = 0.45**
Kit category 7 (nominal range 200–300 μg/L)	0.003	***p*(kit category = 7 | As = 100 μg/L) = 0.2**
Kit category 8 (nominal range 300–500 μg/L)	0.0003	*p*(kit category = 8 | As = 100 μ**g/L**) = 0.04
Kit category 9 (nominal range 500–1000 μg/L)	<0.0001	*p*(kit category = 9 | As = 100 μg/L) < 0.0001

*Note*. Kit categories (5–7) with high probability are highlighted in bold. The conditional probabilities are calculated using Equation 1.

These conditional probability density functions are then used to quantify the probability that a kit measurement falls in any category, correct or incorrect, given a spectroscopic (laboratory) measurement, and hence errors that occur when kit measurements are used to label wells. The probability of assigning blue placards to wells with arsenic between 0 and 10 μg/L (correct assignment) is:

(2) $$P\left(i=\left\{1,\;2\right\}|\theta <10\right)\;=\frac{\sum_{1}^{2}\int_{0}^{10}f_{i}\left(\theta \right)d\theta }{\sum_{1}^{9}\int_{0}^{10}f_{i}\left(\theta \right)d\theta }\;$$

where *i* = 1 and 2 corresponds with nominal kit range of 0–1 and 1–10 μg/L. Similarly, the probability of assigning green or red placards when the accurate measurement is less than 10 μg/L (incorrect assignment) is given by:

(3) $$P\left(i=\left\{3,4,5,6,7,8,9\right\}|\theta <10\right)\;=\frac{\sum_{3}^{9}\int_{0}^{10}\;f_{i}\left(\theta \right)d\theta }{\sum_{1}^{9}\int_{0}^{10}\;f_{i}\left(\theta \right)d\theta }$$

where *i* = 3–9 corresponds to nominal kit categories with range of >10 μg/L (Figure 2).

For wells between 10 and 50 μg/L, the probability of assigning correct (green), false negative (blue) and false positive (red) placard is given by Equations 4–6 respectively:

(4) $$P\left(i=\left\{3,\;4\right\}|10<\;\theta <50\right)\;=\frac{\sum_{3}^{4}\int_{10}^{50}\;f_{i}\left(\theta \right)d\theta }{\sum_{1}^{9}\int_{10}^{50}\;f_{i}\left(\theta \right)d\theta }\;$$

(5) $$P\left(i=\left\{1,\;2\right\}|10<\;\theta <50\right)\;=\frac{\sum_{1}^{2}\int_{10}^{50}\;f_{i}\left(\theta \right)d\theta }{\sum_{1}^{9}\int_{10}^{50}\;f_{i}\left(\theta \right)d\theta }\;$$

(6) $$P\left(i=\left\{5,6,7,8,9\right\}|10<\;\theta <50\right)\;=\frac{\sum_{5}^{9}\int_{10}^{50}\;f_{i}\left(\theta \right)d\theta }{\sum_{1}^{9}\int_{10}^{50}\;f_{i}\left(\theta \right)d\theta }$$

For wells >50 μg/L, the conditional probability of correct (red) and false negative (green or blue) assignments is given by Equations 7 and 8 respectively:

(7) $$P\left(i=\left\{5,\;6,7,8,9\right\}|\theta >50\right)\;=\frac{\sum_{5}^{9}\int_{50}^{\infty }\;f_{i}\left(\theta \right)d\theta }{\sum_{1}^{9}\int_{50}^{\infty }\;f_{i}\left(\theta \right)d\theta }$$

(8) $$P\left(i=\left\{1,2,3,4\right\}|\theta >50\right)\;=\frac{\sum_{1}^{4}\int_{50}^{\infty }\;f_{i}\left(\theta \right)d\theta }{\sum_{1}^{9}\int_{50}^{\infty }\;f_{i}\left(\theta \right)d\theta }\;$$

We also calculated the conditional probability of a well being assigned as safe and unsafe as following:

(9) $$P\left(i=\left\{1,\;2,\;3,\;4\right\}|\;\text{Arsenic}\;=\;\theta \right)\;=\frac{\sum_{1}^{4}f_{i}\left(\theta \right)d\theta }{\sum_{1}^{9}f_{i}\left(\theta \right)d\theta }\;$$

(10) P(i={5,6,7,8,9}|Arsenic=θ)=1−P(i={1,2,3,4}|Arsenic=θ)

### 2.3 Well Switching From Blanket Testing

We use the large data set of accurate arsenic measurements that represents nearly all wells in a 25 km^2^ portion of Araihazar to investigate the efficacy of well‐switching based on different types of measurements and different reported results on pump heads. Because we have calculated the conditional probabilities from the paired data set (describe in previous section), we have the tools to simulate field‐kit measurements from the accurate measurements (see Table 1) without actually having field‐kit measurements for all wells.

We then simulate switching for all wells within 100 m of each other; each household will switch to a better well if it is within 100 m. In Bangladesh and India, the probability of well switching drops with distance to a well (Barnwal et al., 2017; Gelman et al., 2004; Madajewicz et al., 2007; Opar et al., 2007; Pfaff et al., 2017), so that the probability of switching is low (<0.3) if the distance between the unsafe and the safe well is greater than 100 m. In our analysis, we assume that everyone switches from an unsafe well if a safe well is within 100 m.

We evaluated well‐switching for 11 different measurement and reporting scenarios. We judged the effectiveness of each of the 11 plans by calculating the mean reduction in arsenic exposure pre‐and post‐switching. We divide the 11 plans into 3 groups: In Group A, we investigate switching based upon spectrometric (laboratory) measurements; in Group B, switching is based on the simulated kit categories; and in Group C, we investigate the effects of spatial correlation on switching.

If groundwater arsenic is measured accurately, switches will only be to less contaminated wells. However, using inaccurate field kits leads to a variety of poor switches. First, a switch can take place between a correctly identified contaminated well (nominal kit categories 5–9) to an incorrectly identified safe well (bad switching). Second, a switch can take place between a safe well that is incorrectly identified as contaminated to another safe well that is correctly identified as safe (unnecessary switching). Third, a switch can take place between a safe well that is incorrectly identified as unsafe to a contaminated well that is incorrectly identified as safe (very bad switching). Fourth, an unsafe well incorrectly assigned as safe will not switch (missed opportunity for switching). All these possible switching scenarios and their associated probabilities are provided in Table 2. Thus, switching based on field kits can result in unnecessary, bad, or failed switching which is not the case for switching based on laboratory measurements.

**Table 2:** Possible Switching Scenarios Based on the Probability of Correct, and Incorrect Nominal Kit Category Assignments

Type of switching	Probability of switching	Description
Ideal Switching	*p*(*i* = {5,6,7,8,9}|*θ* > 50) × *p*(*i* = {1,2,3,4}|*θ* < 50)	Switched from correctly identified unsafe well to correctly identified safe well
Bad Switching	*p*(*i* = {5,6,7,8,9}|*θ* > 50) × *p*(*i* = {1,2,3,4}|*θ* > 50)	Switched from correctly identified unsafe well to incorrectly identified safe well
Very bad switching	*p*(*i* = {5,6,7,8,9}|*θ* < 50) × *p*(*i* = {1,2,3,4}|*θ* > 50)	Switched from incorrectly identified safe well to incorrectly identified unsafe well
Unnecessary switching	*p*(*i* = {5,6,7,8,9}|*θ* < 50) × *p*(*i* = {1,2,3,4}|*θ* < 50)	Switched from incorrectly identified safe well to correctly identified safe well
Missed switching	*p*(*i* = {1,2,3,4}|*θ* > 50)	False negative identification of unsafe wells. Therefore, no switching

*Note*. *i* is the different kit category and *θ* is the true arsenic concentration. Actual switching only takes place when the well to be switched to lies in a 100‐m radius of the well that is being switched from.

#### 2.3.1 Group A: Switching Based on Laboratory Measurements

Scenario A1 Everyone switches to the well with the lowest arsenic concentration within a 100‐m radius. This ideal but unrealistic plan serves as a point of comparison for more realistic scenarios and provides an upper bound on the possible reduction in exposure.

Scenario A2 We investigate the effects of the modest errors in laboratory measurements of ±10%. This scenario is similar to scenario 1, except that we add a random normal error with a standard deviation of ±10% of the value to each data point. The purpose of this scenario is to quantify the effect of analytical uncertainty on the well switching exercise. We did not add uncertainty to wells with arsenic concentration of 0 μg/L and the concentration of these wells kept at 0 μg/L—primarily because several wells with a measured concentration will have an unrealistic negative concentration after incorporating the uncertainty.

Scenario A3 We consider the effect of labeling wells as categorically safe or unsafe rather than using concentrations. Thus, wells with arsenic concentration >50 and ≤50 μg/L were labeled red and green respectively. Everyone using the red well switches to a green well—if such a well exists within a 100‐m radius.

Scenario A4 We use the three categories, as is the recent practice in Araihazar where the wells were categorized in three categories (van Geen et al., 2014) instead of the two categories used elsewhere in Bangladesh. In Araihazar, wells with arsenic ≤10 μg/L are labeled blue and wells with arsenic with >10 and ≤ 50 μg/L are labeled green, and above 50 are labeled red. Consumers using red wells switch to the nearest blue well (if any was present) in the 100‐m radius of the well. If there were no blue well consumers switched to the nearest green wells. If there was neither a blue nor a green well in the 100‐m radius, the consumers did not switch.

Scenario A5 Here we find the optimal switching concentration such that the mean exposure after switching is the lowest. The decision to label wells >50 μg/L as contaminated and wells with concentration ≤50 μg/L as uncontaminated in Bangladesh was not chosen specifically to optimize health outcomes. For instance, wells with arsenic concentration just below 50 μg/L (such as 45 μg/L) cannot switch to a nearby well with lower arsenic as both would be labeled green. Similarly, in case where there is no safe well in the vicinity of a contaminated well (such as a well with 230 μg/L), the well cannot switch to a nearby less contaminated well (such as a well with arsenic concentration of 60 μg/L) as both would be labeled red and based upon the color it would be impossible for the consumers to know which well is more contaminated. We investigated the arsenic concentration (10–100 μg/L, with an increment of 1 μg/L) below and above which a well is labeled safe (green) and unsafe (red) to find the switching concentration such that the mean exposure post‐switching (based on well labels) is minimum.

#### 2.3.2 Group B: Switching Based on Kit Measurements

In this group of well‐switching simulations, we consider well switching plans based on kit measurements of arsenic concentration by simulating the kit measurements based on statistical model and observations described earlier.

Scenario B1 Here, we consider well switching based on all nine kit categories and using the statistics of categorization errors (Section 2.1) to simulate mis‐categorizations. Consumers of each well switched to the well assigned with the lowest kit category within a 100‐m radius. This plan differs from typical plans that use only two categories.

Scenario B2 Here, we consider the typical approach of labeling wells in only two categories, safe and unsafe. Wells with kit categories of 5 and above (i.e., nominal arsenic range of 50–100 μg/L and above) were labeled red and wells with kit categories of 1–4 (i.e., nominal arsenic range of less than 50 μg/L) were labeled green. Consumers of the red wells switch to the nearest green well (if any such well was present within a 100‐m radius). This is analogous to switching Scenario A3 based on laboratory measurements and represents the commonly practiced switching scenario in Bangladesh.

Scenario B3 Wells were labeled in three colors as has been done in Araihazar (analogous to spectrometric based switching Scenario A4). Wells with kit categories of 1 and 2 were labeled blue, categories 3 and 4 were labeled green and categories 5 and above labeled red. We then assigned residents using red wells to switch to the nearest blue well (if any present) or else switch to the nearest green wells within a 100‐m radius. If there is no blue or green labeled well, residents do not switch.

Scenario B4 Here, we find the category above which the wells should be labeled red and below which it should be labeled green such that the mean exposure post switching is the lowest. Recent practice in Bangladesh has been to label wells that fall in categories 5–9 red. Here, we consider whether this is the optimal threshold for reducing mean exposure post‐switching. For this exercise we evaluated the exposure post well switching for all the nine categories below and above which the well are labeled as safe and unsafe. For example, we compare the scenario when categories 2–9 are labeled as unsafe with the scenario when categories 6–9 are labeled as unsafe.

#### 2.3.3 Group C: Effects of Spatial Autocorrelation in Arsenic Concentrations

In this set of simulations, we consider how the efficacy of well switching is affected by spatial autocorrelations in arsenic concentrations across wells. Where arsenic concentrations are spatially autocorrelated, well switching is limited because contaminated wells are more likely surrounded by contaminated wells and safe wells are surrounded by safe wells. Hence, the possibility of switching depends not only on the identification of safe and unsafe wells but also on spatial pattern of well arsenic concentrations.

**Figure 5:**
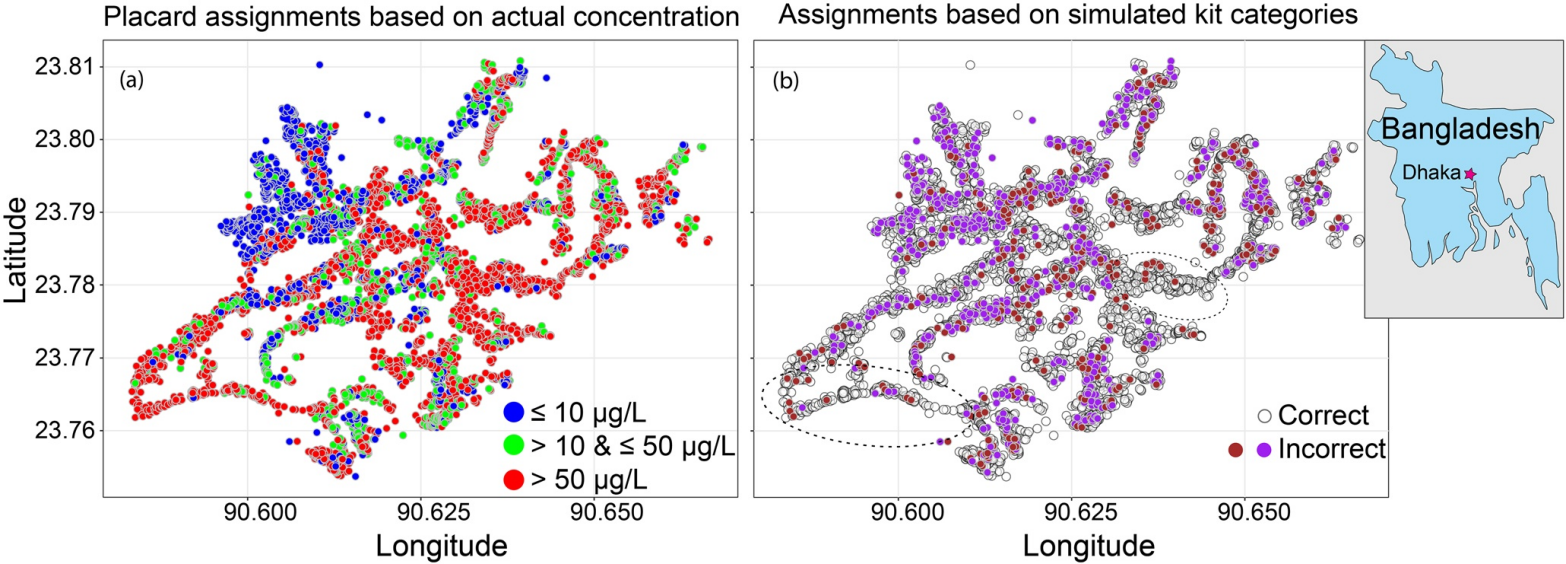
(a) Arsenic concentration of 6595 Araihazar wells measured by spectrometric method. (b) Correct (white circle with black border), and incorrect assignments (purple and brown circles) for simulated categorization based on kit measurements (see Table 2). Wells with As ≤ 10 μg/L that are labeled as green or red and wells with 10 < As ≤ 50 μg/L that are labeled as red are shown in purple. Wells with 10 < As ≤ 50 μg/L that are labeled as blue and wells with As > 50 labeled as blue and green are shown in brown. Two regions with large proportion of correct assignments are highlighted in black ellipses. Inset: Map of Bangladesh. Araihazar is 30 km east of Dhaka (pink star).

Distribution of arsenic in Araihazar are weakly spatially autocorrelated at small scales and contains some larger scale features, particularly a large cluster of low arsenic wells in the northwestern part of the district (Figure 5). To investigate the impact of these patterns on the effectiveness of well switching, we applied two hypothetical switching scenarios that removed spatial autocorrelation. We randomly reassigned each well an arsenic concentration (and the corresponding simulated kit category) from the distribution of the 6595 wells (without replacement). Subsequently, we simulated well switching based upon the reassigned arsenic concentration to each well.

Scenario C1 Here, we consider the effect of spatial autocorrelation when measurements are accurate. This is analogous to Scenario A1 except that the distribution of arsenic is not spatially autocorrelated.

Scenario C2 Finally, we consider here the effect of spatial autocorrelation when kit measurements are used. Analogous to Scenario B1, we simulate switching based upon the reassigned simulated kit categories except that the distribution of arsenic is not spatially autocorrelated.

## 3 Results

### 3.1 Errors in Assignment of Wells to Safe and Unsafe Categories

**Figure 6:**
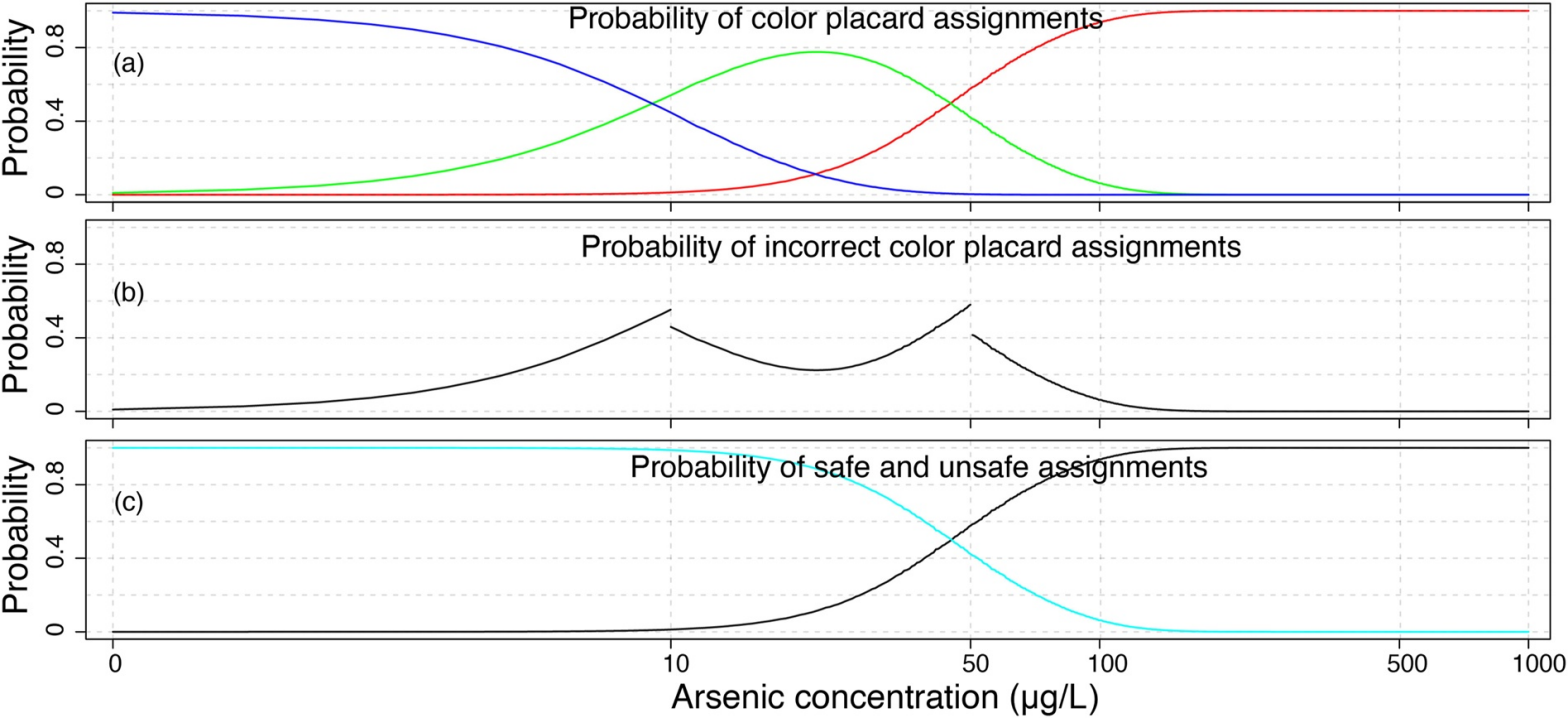
(a) Probability of assigning the different color placards (blue, green and red) from kit measurements of arsenic as a function of arsenic concentration. (b) Probability of assigning incorrect color placard as a function of arsenic concentration. For wells with As ≤ 10 μg/L, probability of incorrect assignment is defined as the sum of assigning green and red placards. For well with 10 < As ≤ 50 μg/L, probability of incorrect assignment is defined as the sum of assigning blue and red placards. For wells with As > 50 μg/L, probability of incorrect assignment is defined as the sum of assigning blue and green placards. (c) Probability of assigning a well as safe (light blue) and unsafe (black) as a function of arsenic concentration.

Mis‐categorization of wells is most likely where the arsenic concentration is close to the threshold, and the probability of error falls off rapidly for concentrations that are far from thresholds (Figure 6). Thus, extremely contaminated wells are unlikely to be classified as safe by the field kits. For example, for arsenic concentrations above 200 μg/L, the probability of incorrectly assigning a well as safe (blue or green placard), was very small (<0.001, Figure 6).

K‐S tests resulted in the following best fits to the distribution of concentrations within each kit category. The Gamma distribution was the best fitting parametric function for the accurate arsenic measurements for categories 3–9. For categories 1 and 2, Weibull and exponential distributions, respectively were the best fitting functions (Figure 4). For kit categories 6–9, the normal distribution was also a good fit to the accurate measurements, however we chose the gamma distribution as it is positively defined, and arsenic concentrations are also ≥0 μg/L.

### 3.2 Well Switching Based on Accurate Arsenic Data (Group A)

The first group of scenarios (group A) contains well‐switching simulations based on accurate measurements (Table 3). Scenario A1 is the ideal base case: switching is based on continuous accurate arsenic data. In this case, 84% of consumers reduce their arsenic exposure by switching and the mean arsenic exposure of the residents decreased from 134 μg/L pre‐switching to 17 μg/L after switching. Scenario A2 investigates the impact of the analytical uncertainty in accurate laboratory measurements of arsenic on well‐switching. The reduction in arsenic exposure was similar to Scenario A1 suggesting that analytical uncertainty in laboratory measurements has a negligible influence on the outcome of well switching.

**Table 3:**
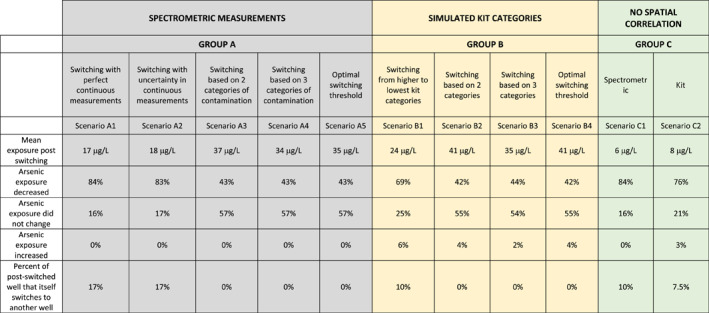
Summary Statistics of Exposure Post Switching and Percentage of Consumers Experiencing Change in Arsenic Exposure for the Different Switching Scenarios

*Note*. Before switching the exposure is the average arsenic concentration across wells 134 μg/L. Scenarios B2 and B4 have same values for the different rows as the optimal threshold (B4) for switching between uncontaminated and contaminated (red and green) wells is observed when wells with kit categories 1–4 are labeled green and categories 5–9 are labeled red which is same as scenario B2.

Scenarios A3 and A4 investigate the impact of categorizing wells based on accurate laboratory measurement. After sorting wells into two categories, red wells (As > 50 μg/L) and green wells (As ≤ 50 μg/L), only 43% of the residents lowered their arsenic concentration and the mean exposure post well switching was 37 μg/L. Fewer switches occurred than in Scenario A1 because no switching occurs between wells with As ≤ 50 μg/L. In Scenario A4 wells were labeled in three categories and again 43% of the residents switched to lower arsenic concentration wells and the mean exposure post well switching was 35 μg/L– only slightly lower than under Scenario A3. A comparison of Scenarios A3 and A4 suggests that the fraction of residents that lower their arsenic exposure is similar when the wells are grouped in two or three color categories, however, the net reduction in arsenic exposure for three groups is slightly better because consumers can switch to wells with low arsenic (≤10 μg/L, blue wells) where possible. Since wells with As ≤ 10 μg/L are mostly concentrated in the northwestern part of Araihazar (Figure 5), the decrease in arsenic exposure by labeling the wells in three categories was limited; however, if the wells with As ≤ 10 μg/L were truly randomly distributed the decrease would have been higher (see Scenarios C1 and C2).

**Figure 7:**
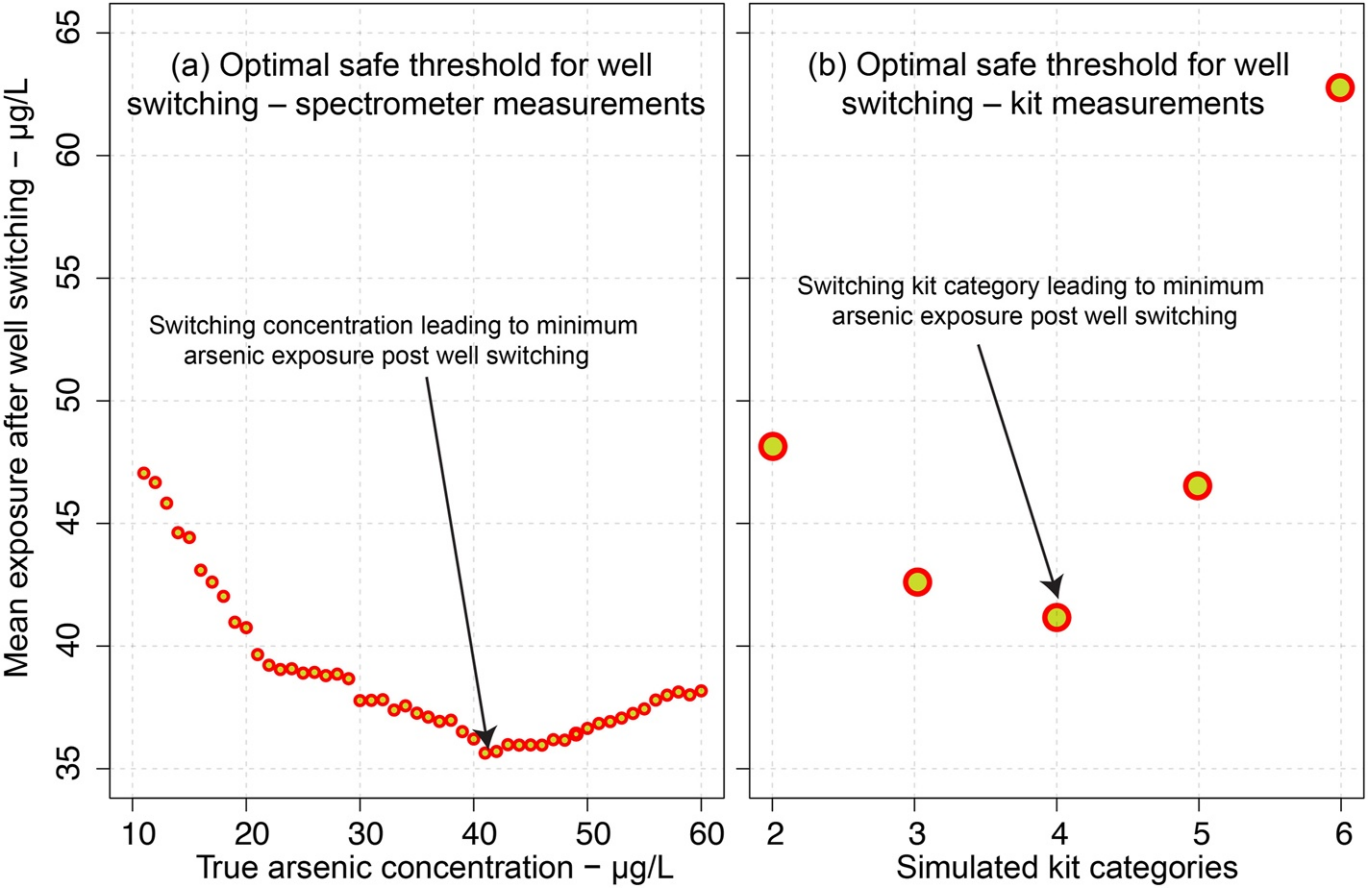
(a) Mean exposure post switching for different “safe” thresholds. The minimum exposure is at 41 μg/L. (b) Mean exposure post switching based on categorical kit measurements. The minimum exposure post switching is observed if wells with categories 1–4 are labeled green and wells with categories 5–9 are labeled red.

The optimal threshold concentration that minimizes mean exposure (Scenario A5) is 41 μg/L (Figure 7a), producing a post‐switching mean exposure of 35 μg/L. This value is only 9 μg/L lower than the mean exposure at the 50 μg/L cutoff that is currently used in Bangladesh (Scenario A3).

### 3.3 Well Switching Based on Simulated Kit Categories (Group B)

In this group of scenarios, we use simulated kit measurements to assess the impact of kit measurement errors. Unlike accurate measurements, kit measurements lead to some switches from lower to higher arsenic concentrations (Table 3, second row from the bottom). Scenario B1 describes the results when all kit categories are used to label wells. With these nine categorical labels, 69% of the consumers reduce their arsenic exposure and 25% of consumers keep the same level of exposure. Because of the inaccuracies of kit measurements, exposure increases for 6% of the consumers. The mean arsenic exposure of the residents was 24 μg/L after well switching.

Scenario B2 describes the typical case across Bangladesh: kit measurements are used to categorize wells as safe or unsafe with a nominal threshold of 50 μg/L. In this case fewer (42%) of the residents lower their arsenic concentration, 54% of residents have the same level of exposure and 4% of the residents increase their exposure. The mean exposure post well switching was 41 μg/L, still a large reduction from the average across wells of 134 μg/L.

Scenario B3 considers the atypical approach used in Araihazar: three categories of wells (blue, green and red), rather than just two. The fraction of residents who reduced their exposure was higher than Scenario B2 (Table 3) and the exposure was reduced to 35 μg/L, about 6 μg/L lower than with two categories, Scenario B2.

The optimal threshold category for switching is the same as what is currently used in Bangladesh to assign the wells green and red placards. Therefore, for minimum arsenic exposure post‐switching kit categories 1–4 should be labeled green and wells with kit categories 5 and above should be labeled red (Figure 7b).

### 3.4 Well Switching Based on Randomization of Arsenic Concentration (Group C)

Group C scenarios are entirely hypothetical, and both consider the effects of spatial autocorrelation across wells on well switching by erasing this correlation. Scenario C1 considers the case of accurate measurements and Scenario C2 considers kit measurements. In both scenarios well switching becomes extremely effective: the exposure drops to 6 and 8 μg/L when arsenic concentrations are random in space because many more households have neighboring wells to switch to (84% and 76%, Table 3).

## 4 Discussion

### 4.1 Is Well Switching Useful?

Exposure to high level of arsenic in drinking water is still pervasive in South Asia with more than 40 million people exposed to high level of arsenic in drinking water in Bangladesh alone (Jamil et al., 2019). Several strategies have been proposed in the past two decades to reduce arsenic exposure in drinking water including filtration of pond and surface water, removal of arsenic at the household level using purification filters, community filtration systems, rainwater harvesting and well switching (Ahmed et al., 2006). Each of these methods has its own problems. Sand filtration is unsuitable due to high concentration of fecal contaminants in surface water (Howard et al., 2006) and the inability of these filters to remove them. Similarly, high cost of household filters and regular maintenance of community filtration processes has led to their limited success in reducing population level arsenic exposure (Krupoff et al., 2020). A recent analysis by Jamil et al. (2019) suggests that well testing and subsequent switching leads to the largest decrease in population level arsenic exposure and is economically the most viable solution for reducing population level arsenic exposure. The total cost for kit based well testing ($1) and subsequent switching is significantly lower than the cost per person associated with installing low arsenic deep wells ($143) and operating a treated piped water supply system ($158).

Although well testing by kit measurements are economically feasible and logistically viable, they have been criticized for their lack of accuracy and precision (Jakariya et al., 2007; Reddy et al., 2020). An important question to ask is what are the pros and cons of using field kits for well switching, how do they compare with accurate but expensive laboratory‐based measurements for well switching and at a community level it is a sustainable option to recommend for large scale well switching?

For all the switching scenarios presented here, the mean exposure post switching (Table 3) was substantially lower than the pre‐switching arsenic exposure of 134 μg/L. Excluding the hypothetical scenarios where the spatial distribution of well‐water arsenic was randomized (Scenarios C1 and C2), the net arsenic exposure post switching in Araihazar was 3–7 times lower than the exposure before switching. Even for the simulated kit‐based switching scenarios, the net decrease in arsenic exposure was 3–5 times lower. The most important outcome of well switching (in all scenarios) was the ability to reduce the exposure of consumers using highly contaminated wells (>100 μg/L) to significantly lower levels. Even for the least effective scenario (B2), the average exposure for people exposed to wells with arsenic >100 μg/L reduced from 214 to 74 μg/L.

The analysis presented here complements the cost–benefit analysis by Jamil et al. (2019) and provides support that well switching based on kit measurements is not only economically feasible but it also very effective in reducing population level arsenic exposure. Therefore, even though kit measurements can be inaccurate, they can lead to significant decrease in arsenic exposure at community level. The higher accuracy laboratory measurements render them only marginally better in terms of reducing arsenic exposure and require samples to be taken to the laboratory and results back to the users, which could be a logistic challenge and will undoubtedly increase costs.

Notwithstanding the net decrease in arsenic following laboratory and kit‐based measurements, it has been well documented that the well switching is not complete due to multiple factors including the distance between safe and unsafe wells and socioeconomic factors. In India, Barnwal et al. (2017) showed that the probability of well switching decreases rapidly as the distance between the safe and the unsafe well increases—if the distance between the safe and unsafe well is ≤10 m the probability of well switching is ∼0.4 and if the distance is >100 m, the probability is <0.25. In Bangladesh, Madajewicz et al. (2007) reported that in Araihazar, 60% of the people who realized they were using contaminated well switched to a safe well within 1 year. These are high response levels, even if the maximum level of exposure reduction was not achieved and household knew that they were using contaminated water. One reason may be that many households with a low arsenic well might not be willing to share their wells with their neighbors; households with lower socioeconomic status find it more challenging to switch to safe wells (Madajewicz et al., 2007). The implication is that there is considerable potential for additional switching and more attention should be paid to ways of encouraging well switching and sharing among neighbors.

### 4.2 Comparing Laboratory‐ and Kit‐Based Switching

The arsenic exposure after well switching and the proportion of households managing to switch were similar based on laboratory and kit measurements (Table 3). There are three major factors behind this surprisingly good outcome for kit‐based switching. First is the ability of the kits to correctly identify the uncontaminated (arsenic ≤10 μg/L) and highly contaminated wells (>100 μg/L) with a high degree of accuracy (>0.95, Figure 6a). Second, the distribution of groundwater arsenic in Araihazar is non‐normal: 30% of wells contain ≤10 μg/L arsenic, 34% of the wells contain between 10 and 100 μg/L, and 36% contain >100 μg/L arsenic. This resulted in the accurate labeling by the kit for approximately 66% of the wells with arsenic ≤10 μg/L or >100 μg/L. Indeed, most mis‐categorizations (false positive and false negative) was observed for the 34% of the wells with true concentration between 10 and 100 μg/L (Figure 6b). Third the overall degree of spatial autocorrelation in the distribution of groundwater arsenic was low—although for some pockets arsenic concentration was strongly autocorrelated (Figure 5). Therefore, contaminated and uncontaminated wells (Figure 5) were always in close proximity resulting in large amount of switching.

The comparative analysis also highlights three major limitations of switching based on kit measurements. First, is the continued exposure of consumers using contaminated wells that were incorrectly assigned as safe. More than 22% of the wells with arsenic between 50 and 75 μg/L were assigned a kit category between 1 and 4 (i.e., blue or green placard). This prevented them from switching to a nearby safe (or less contaminated) well. Second, more than 40% of the wells with arsenic between 20 and 50 μg/L were incorrectly assigned kit category of 5 and above (i.e., red placard). This resulted in unnecessary switching by consumers using these wells. In general, most consumers switched to a correctly categorized nearby uncontaminated well, however, some consumers switched to a well with higher arsenic concentration than their original well (bad switching). This led to an increase in arsenic exposure for 4% and 2% of the consumers in Scenarios B2 and B3 respectively. Although this switching is extremely undesirable, the net increase in arsenic exposure of consumers experiencing bad switching was not high (15 μg/L for Scenario B2).

### 4.3 Should Wells Be Grouped in 3 Color Categories?

Typically, well switching programs use color placards placed on the well. Across Bangladesh, wells have been labeled green (≤50 μg/L) or red (>50 μg/L). However, more recently in the Araihazar subdistrict, wells were labeled blue (≤10 μg/L), green (>10–50 μg/L), or red (>50 μg/L). It is important to ask if there is an added advantage in labeling wells in three color categories. Our analysis suggests that the proportion of population switching from contaminated well to uncontaminated well based on two‐ or three‐color placards are the same (compare Scenarios A3, A4, B2 and B3, Table 3). Based on laboratory measurements the mean exposure post switching is comparable for both the scenarios (37 and 34 μg/L based on two‐ and three‐color placard categories, respectively). The difference based on the simulated kit categories was slightly higher but not very large (mean exposure post switching was 41 and 35 μg/L based on two‐ and three‐color placard categories, respectively). In Araihazar, it appears that grouping the wells in 3 categories is only slightly more beneficial. The main factor driving this pattern is the clustering of majority of the wells with arsenic <10 μg/L (i.e., blue wells) in the northwestern part of the district. Therefore, the benefit provided by grouping the wells in 3 color categories only helped a minority of the consumers. This could be different in other regions of Bangladesh with a more spatially mixed distribution of arsenic in wells.

It is worth noting that the mean reduction in exposure based on actual concentration (and actual kit categories) was almost twice as low than those based on color categories (compare Scenarios A1 and A3, and B1 and B2 respectively, Table 3). Thus, if placards placed on the well also included the concentration (or the kit categories when field kits are used), the possible reduction in exposure could be substantially higher. In the original HEALS study, actual arsenic concentration was included on the well placard (Chen et al., 2007) and 58% of the 6,512 participants using contaminated wells (As ≥ 50 μg/L) switched to other wells. Therefore, including arsenic concentration (or the kit category) on the color placard might bear an additional cost but could lead to higher switching rates (Madajewicz et al., 2007). Since well‐switching is voluntary, providing the actual concentration (or the kit categories) would also provide the consumers more freedom in deciding if they want to switch and which well to switch to.

According to the Bangladesh Arsenic Mitigation and Water Supply Program (BAMWSP) survey of 2000–2005, the number of wells with As > 50 μg/L in Araihazar and across Bangladesh are comparable (29% and 32% respectively). Additionally, the spatial heterogeneity–which is critical for effective well switching–across the upazilas (sub‐districts) in Bangladesh is also comparable to Araihazar (Jamil et al., 2019). Although the testing under BAMWSP underestimated the number of high As wells (van Geen et al., 2005), the similarity in the number of well with As > 50 μg/L and in the spatial heterogeneity of groundwater arsenic concentration suggest that the findings presented here are relevant across the country. The number of wells has increased rapidly in Bangladesh in the last 10 years (Jamil et al., 2019), and most of these wells are untested for As. After the end of BAMWSP campaign in 2005 there has been no blanket testing in Bangladesh and the current nationwide arsenic exposure in Bangladesh is unquantified. Our analysis suggests that a nationwide blanket testing followed by widescale well switching has the potential to reduce mean arsenic exposure to concentrations lower than the current Bangladesh standard of 50 μg/L for most of the districts.

### 4.4 Is 50 μg/L the Optimal Level for Labeling Unsafe Wells?

From a health perspective, various drinking water standards or the WHO guideline for arsenic are somewhat arbitrary. The WHO guideline of 10 μg/L is most widely referred to globally (Ahmad & Bhattacharya, 2019), however, the standard for arsenic in drinking water also varies regionally. In the Netherlands, for instance, the voluntary target of arsenic in drinking water is <1 μg/L (Ahmad et al., 2020). In the United States, the EPA lowered the drinking water standard for arsenic from 50 to 10 μg/L in 2001, but the state of New Jersey has lowered it to 5 μg/L. In Bangladesh and Pakistan, the standard for arsenic in drinking water is still 50 μg/L. India recently changed the standard for arsenic in drinking water to 10 μg/L. There is a continuum in toxicity across the range of arsenic concentrations and health effects do not suddenly appear with an increase from 9 to 11 μg/L or from 45 to 55 μg/L.

In Bangladesh, wells with arsenic >50 μg/L and/or wells with observed kit categories of 5–9 are considered contaminated and the well with arsenic <50 μg/L are considered safe. Consequently, users of wells with >50 μg/L arsenic (i.e., observed field kit categories of 5–9) have been encouraged by the Bangladeshi government to switch to the nearby safe wells. To our knowledge, the safe threshold of 50 μg/L in Bangladesh was not chosen to minimize arsenic exposure post well switching. As discussed in Section 1.2, the optimal threshold values for well switching are not necessarily the concentration that has been deemed safe to drink and the optimal switching concentration can vary from region to region. Our analysis shows that the maximum reduction in arsenic exposure is observed if wells with As > 41 μg/L and wells with observed kit categories of 5–9 are considered as unsafe (Figure 7). This suggests that the currently used criterion of switching consumers using wells with arsenic >50 μg/L (and observed kit categories of 5–9) happens to be fairly close to the optimal switching concentration. For switching based on 50 μg/L threshold, the mean exposure was always lower than 41 μg/L. In comparison, if all the wells with arsenic >10 μg/L are labeled as contaminated the mean exposure post switching would be 46 μg/L or greater. Similarly, labeling wells with simulated kit category of 2–9 as contaminated would result in a mean exposure of 48 μg/L after well switching. Therefore, from a switching perspective, labeling well with arsenic >50 μg/L as unsafe would lead to higher reduction in exposure post switching than labeling wells with arsenic >10 μg/L as unsafe.

### 4.5 Are Kit Tests Preferable to Laboratory Measurements?

Laboratory measurements of arsenic concentration are more accurate than field kit measurements and so provide more reliable data for labeling safe and unsafe wells. However, the use of imprecise field kits (Reddy et al., 2020) should be considered in a broader framework where a major motivating factor in using the less accurate kit‐based measurements are their cost‐effectiveness, availability on the spot, and independence from expensive spectrometric instruments that are often unavailable in developing countries. Funds available at the village level are often limited. We evaluated whether, given a certain amount of funding, it is more fruitful to test a small number of wells using the more accurate but expensive spectrometric measurements or test a large number of wells using kits.

We address this question as a case study, assuming that a village is allocated $2000 to test arsenic in the groundwater wells. With that amount, the village can accurately measure arsenic in 200 wells in the laboratory ($10 per sample (Gelman et al., 2004),) or measure 2000 wells albeit with less accuracy using field kit ($1 per sample (Ahmed et al., 2006)). If we assume that the distribution of arsenic concentration in this village is similar to Araihazar, then kit measurements could lead to a possible reduction of arsenic exposure for 6000 consumers (assuming 10 consumers per well) from >50 to ≤50 μg/L. However, 140 people would most likely experience an increase in arsenic exposure due to the uncertainties associated with kit measurements. In contrast, laboratory measurements would lead to a decrease in arsenic exposure for 800 people from >50 to 50 μg/L with no one experiencing an increase in exposure. Thus, with a limited budget, kit measurements can reduce arsenic exposure for almost 8 times more people than laboratory measurements. However, this also led to an increased exposure of approximately 15 out of every 1000 people to higher levels of arsenic. This does lead to a moral dilemma from having to choose between a laboratory method that helps a small fraction of the population but does not adversely affect a single person in the population and a field‐based method that helps a larger proportion of the population but could increase the arsenic exposure of a small proportion of the population.

From a utilitarian perspective that considers benefits to the population overall, the second scenario is clearly preferable. If increased arsenic exposures for a small proportion of the population is acceptable, then the case study provides compelling evidence that large quantities of lower‐grade and imperfect kit‐based measurements may be more effective in mitigating arsenic exposure than a small number of more precise spectrometric measurements—at least for places with an arsenic distribution similar to Araihazar.

### 4.6 Promoting Well‐Testing to Mitigate Arsenic Exposure

It is estimated that arsenic related mortality will cost Bangladesh almost $12.5 billion in the next 20 years (Flanagan et al., 2012). Arsenic exposure from drinking well‐water has also been shown to reduce household income substantially (Pitt et al., 2021). This helps explain why reducing arsenic in drinking water arsenic is an important goal of the Bangladesh government. Currently, well switching is the most cost‐effective and is a scalable solution for reducing arsenic in drinking water across Bangladesh. Our analysis suggests that imprecise kit measurements can reduce arsenic exposure of more than 85% of the population of Araihazar to ≤50 μg/L (Bangladesh standard) if well switching was complete. However, the well switching rate is moderate (varies between 30% and 60%) due to multiple factors (Barnwal et al., 2017; Jamil et al., 2019; Madajewicz et al., 2007) and the effective exposure at the population level remains high.

A large‐scale decrease in arsenic exposure can only be achieved if wells are extensively tested and the results are shared with the households. Selling tests is not an option as it has already been shown that the demand for a field‐kit test drop rapidly at any price that could potentially sustain a commercial testing service (Barnwal et al., 2017; Tarozzi et al., 2020). Therefore, testing should be offered free, and the results should be shared with all households.

Krupoff et al. (2020) provided an analysis of well switching in Bangladesh from the perspective of the social sciences and suggested multiple reasons for modest switching rates and provided recommendations for increasing well switching rates in Bangladesh. First, the low rates of well switching could be the failure to provide the information to the consumers. Tests are commonly conducted by representatives who leave the village after performing the test leaving little opportunity to reinforce the information. In this regard training community members to perform arsenic measurements locally and constantly reinforce the information might be more help promote well switching. Providing monetary compensations might increase the commitment from the community members involved in testing and promoting well switching (BenYishay & Mobarak, 2019). Also, important would be to develop a mechanism that promotes well sharing—such as combining testing with a community commitment (Inauen et al., 2014).

### 4.7 Low Spatial Autocorrelation Is Essential for Effective Well Switching

The degree of spatial autocorrelation in arsenic concentration of the 6595 wells in Araihazar is low (Moran's *I* = 0.1, *Z* = 0.0006, *p* < 0.05), however there is a large cluster of well with arsenic <10 μg/L in the northwest region and arsenic >50 μg/L in the southwest region. After randomizing the arsenic concentration in the well (Moran's *I* = −0.0007, *Z* = −0.01, *p* = 0.35 after randomization), the mean exposure post switching decreased to 6 μg/L (using accurate spectrometric data) and 8 μg/L (using simulated kit categories, Table 3). The large decrease after randomization suggests that vast majority of the wells managed to switch to a blue well (arsenic <10 μg/L) in their vicinity. This highlights the importance of spatial autocorrelation in well switching exercise—in Araihazar even though the degree of spatial autocorrelation is low, yet some consumers (around 15%) were unable to switch due to lack of uncontaminated wells in their 100‐m radius. In villages where groundwater arsenic concentration is highly spatially autocorrelated, the effectiveness of well switching would be fairly limited, however if the spatial autocorrelation in arsenic concentration is weak well switching exercise would be fairly effective. Across much of Bangladesh, spatial correlation in groundwater arsenic is weak (Gelman et al., 2004; Yu et al., 2003) providing strength to well switching as an effective approach to reducing arsenic exposure in drinking water.

## 5 Conclusions

Using simple distance‐based analyses, we have shown that even with its limited accuracy, the mean exposure post‐switching based on kit measurements is not much higher than exposure post‐switching based on laboratory measurements. If a slight increase in arsenic exposure of a small proportion of the population is acceptable, then kits provide a cheap alternative of reducing arsenic exposure for the overall population, especially if actual results are reported instead of merely safe/unsafe. Widespread well switching could significantly reduce arsenic exposure in Bangladesh in the short term and until more sustainable solutions are developed.

## Conflict of Interest

The authors declare no conflicts of interest relevant to this study.

## Data Availability

Data sets analyzed in this study are available at Hydroshare (Groundwater arsenic measurements from Araihazar, Bangladesh) and can be downloaded freely using the following link (https://doi.org/10.4211/hs.8e1373d87419447c945625af13f0a2ea).
